# Executive Functioning, Diabetes Distress, and Diabetes Management Among Adolescents With Type 1 Diabetes: Youth and Parent Perspectives

**DOI:** 10.1155/pedi/7036544

**Published:** 2025-02-27

**Authors:** Allison Choe, Emily M. Fredericks, Dana Albright, Joyce M. Lee, Julie M. Sturza, Hurley O. Riley, Niko Kaciroti, Katherine W. Bauer, Alison L. Miller

**Affiliations:** ^1^Department of Psychology, University of Michigan, East Hall 1004 530 Church Street, Ann Arbor 48109, Michigan, USA; ^2^Department of Pediatrics, Michigan Medicine, University of Michigan, 1540 E Hospital Dr, Ann Arbor 48109, Michigan, USA; ^3^Parkview Mirro Center for Research and Innovation, 10622 Parkview Plaza Dr, Fort Wayne 46845, Indiana, USA; ^4^Department of Health Behavior and Health Equity, School of Public Health, University of Michigan, 1415 Washington Heights, Ann Arbor 48109, Michigan, USA; ^5^Department of Nutritional Sciences, School of Public Health, University of Michigan, 1415 Washington Heights, Ann Arbor 48109, Michigan, USA

**Keywords:** adolescence, diabetes-related distress, EF, executive functioning, glycemic management, T1D, type 1 diabetes

## Abstract

**Objective:** Most adolescents with Type 1 Diabetes (T1D) do not achieve recommended glycemic targets, placing them at risk for long-term complications. Executive functioning (EF), or the cognitive processes that support goal-directed action and management of behavior, emotion, and cognition, is proposed to support effective T1D management and contribute to glycemic stability. We sought to examine associations of EF with T1D management behaviors and diabetes-related distress in adolescents with T1D.

**Methods:** Participants were 13–17-year-olds (*M* = 15.44, *SD* = 1.38 years) from a randomized controlled trial (*N* = 88). We conducted secondary analyses of preintervention data. Youth and their parents each reported on youth EF (Behavior Rating Inventory of Executive Functioning; BRIEF) and T1D management behaviors (Self-Care Inventory-Revised; SCI-R), parents reported on responsibility for T1D management (Diabetes Family Responsibility Questionnaire; DFRQ), and youth reported on their diabetes-related distress (Problem Areas In Diabetes-Teen; PAID-T). Youth also completed performance-based measures of EF.

**Results:** Questionnaire-based and performance-based EF measures were generally unrelated. Regression analysis showed that youth self-reported EF predicted youth-reported T1D management (SCI-R) and diabetes distress (PAID-T) outcomes, and parent-reported youth EF predicted parent-reported T1D management behaviors, such that greater EF difficulties predicted suboptimal management and greater diabetes-related distress (youth PAID-T *β*: 0.41, *p*  < 0.01; youth SCI-R *β*: −0.40, *p*  < 0.01; parent SCI-R *β:* −0.33, *p*  < 0.01). Older child age and poorer performance-based EF also predicted greater youth responsibility for T1D management (age *β*: 0.43,*p*  < 0.01; EF reaction time *β*: 0.23,*p* < 0.05; EF accuracy *β*: −0.23, *p* < 0.05).

**Conclusions:** Youth EF may shape which adolescents are at increased risk for suboptimal T1D management as well as diabetes distress; understanding EF challenges may help guide T1D family management across this developmental period. Implications for EF measurement approaches in youth are also discussed.

**Trial Registration:** ClinicalTrials.gov identifier: NCT03688919

## 1. Executive Functioning, Diabetes Distress, and Diabetes Management Among Adolescents With Type 1 Diabetes: Youth and Parent Perspectives

Managing Type 1 diabetes (T1D), a chronic, autoimmune condition often diagnosed in childhood or adolescence is complex. For example, everyday tasks often include monitoring blood glucose, administering insulin through injections or a pump, tracking carbohydrate intake, exercising, organizing supplies, and managing hypo- and hyperglycemia [1].

The daily demands of managing T1D can pose significant challenges during adolescence and contribute to difficulties with glycemic management [2]. T1D Exchange Quality Improvement Collaborative data revealed that only 17.7% of youth between 13 and 18 years old met the American Diabetes Association's targets for hemoglobin A1c (HbA1c), which is currently <7% [3]. Suboptimal T1D management during adolescence may be attributed to several factors, including the transition from parent/caregiver management of T1D to self-management, increased insulin resistance resulting from hormonal changes, and psychosocial factors, including diabetes distress [4]. Although adolescents' T1D management behaviors such as adherence to regimens and level of parent involvement in daily management tasks can be shaped by environmental influences, it is also important to consider how individual factors may shape management in order to provide individualized supports.

Executive functioning (EF) skills, or the set of interrelated cognitive processes that support goal-directed action and modulation of cognition, emotions, and behaviors [5], may influence an adolescent's ability to manage T1D [6]. EF skills include both top-down and bottom-up processes that operate to regulate behavior, emotion, and cognition on varying timescales from seconds to days, weeks, or years (see Nigg 2017 and Diamond, 2013). For example, the EF skill of inhibitory control can enable an individual to ignore distractions at the moment, working memory skills can allow an individual to recall goals, and higher-order planning skills can allow the achievement of such goals [7]. Prior research has identified inhibitory control, working memory, and planning as key EF skills that can help youth navigate the many daily tasks and frequent adaptations required to manage T1D [8, 9].

Beyond modulating attention and cognition to support managing T1D tasks, EF skills can shape emotion regulation by supporting an individual's capacity to appraise emotional situations and express emotions in an adaptive manner [10]. Among adolescents, EF difficulties have been associated with the use of maladaptive emotion regulation strategies (e.g., suppression, avoidance) and higher depressive symptoms [11–13]. Adolescents with T1D may experience feelings of frustration, fear, or overwhelm due to the challenges of living with diabetes [14]. Such “diabetes distress” is common among youth with T1D [2 ], with studies reporting that one of three youth endorse significant diabetes-related distress [14]. Greater diabetes distress has been uniquely associated with poorer glycemic control and management of T1D in youth [15].

EF skills that support emotion regulation by building emotional awareness and promoting intentional rather than impulsive responses to negative emotions may help youth manage their diabetes distress. One study of adolescents found that emotion regulation characterized by avoidant coping was associated with greater diabetes distress and poorer glycemic control [16]. An intervention to promote T1D resilience in youth engaged higher-order EF skills such as perspective-taking and reflection and found significant [17] and sustained reductions in diabetes distress over 3 years [18]. Yet, no studies have examined how EF skills relate to diabetes distress in youth.

In contrast to diabetes distress, prior studies have examined associations between EF and T1D management behaviors. In a 2014 review, weaker adolescent EF as measured by parent and/or adolescent report was associated with greater T1D management difficulty and suboptimal glycemic stability [8]. A 2021 review by Ding et al. [19] identified mixed evidence for associations between EF and T1D self-management among adolescents, with results varying based on how EF was measured [19]. Across the 15 studies reviewed, EF was either measured using performance-based tasks or questionnaires answered by parents and/or adolescents (most often the Behavior Rating Inventory of Executive Functioning, or BRIEF; most often the Behavior Rating Inventory of Executive Functioning, or BRIEF; [20]). The association between weaker EF and less consistent T1D management was more pronounced in studies where EF was measured using questionnaire-based versus performance-based measures. Ding et al. [19] proposed that a possible reason for this discrepancy could be that performance-based tasks may capture EF skills that operate on a shorter timescale to control behavior, emotion, or cognition at the moment such as inhibitory control and working memory versus higher-order EF skills such as planning that are needed for focusing on long-term goals [5]. Yet, they also noted that only four studies used both performance-based and questionnaire-based measures of EF, with three of these including only high school seniors [21–23]. As both EF and T1D management behaviors change significantly across adolescence, it may be helpful to consider how EF skills demonstrated in performance-based tasks relate to T1D management above and beyond parent and/or youth reports across a broader age range [5].

EF skills emerge across childhood and adolescence and develop at different rates; for example, inhibitory control skills start to develop in early childhood whereas more “top-down”, higher-order skills like planning begin to mature across adolescence [24]. One study of 65 parent–adolescent dyads reviewed by Ding et al. [19] included 13–17 year-olds, but only parents reported on youth EF, not the youth themselves [25]. Yet, parent and youth perceptions of both EF and T1D management may differ, and some have found stronger associations between parent-reported compared to youth-reported EF and T1D-related outcomes [6]. Including perspectives from younger adolescents in this research is important as they are in a period of rapid changes in hormones as well as brain maturation, with implications for emotion regulation and EF development [24]. Gathering perspectives on EF, T1D management, and diabetes distress from youth and parents across this period of development can help us better understand the role of EF in the overall picture of T1D management during this time of transition.

### 1.1. Current Study

The goals of the current study were to examine how varied measures of EF relate to T1D management behaviors and diabetes distress during adolescence. Specifically, we examine whether youth reports, parent reports, and performance-based measures of EF are associated with parent and youth-reported T1D management behaviors, parent-reported responsibility for the management of T1D in the family, and youth-reported diabetes distress. We also consider how parent, youth, and performance-based assessments interrelate. We hypothesize that greater EF difficulties will be associated with suboptimal T1D management and greater diabetes distress. We also expect that EF difficulties will correspond to more parent responsibility for the management of T1D. Furthermore, we predict that parent and youth reports of EF will be more strongly associated with T1D management and diabetes distress than performance-based EF.

## 2. Methods

### 2.1. Participants and Procedures

This study uses preintervention, cross-sectional data from a randomized controlled trial, Adolescent Interventions to Manage Self-Regulation of T1D (AIMS-T1D [Blinded for Review] data available on request), which began data collection in May 2019. Participant and caregiver (97% parents, 8% were fathers; henceforth “parents”) dyads (dyad *N* = 88) were recruited from pediatric endocrinology clinics at a university-affiliated medical center and had to meet the following eligibility criteria at intake to participate in both the RCT and current analysis: the adolescent (a) was 13–17 years old; (b) had been diagnosed with T1D for at least 6 months; (c) had HbA1c ≥ 7.0%; (d) resided with the participating parent at least 50% of the time; (e) had regular access to a smartphone and Wi-Fi; (f) had no psychiatric or cognitive conditions that would hinder their ability to participate; and (g) self-reported sufficient fluency and comfort in English (parents were asked whether they felt comfortable speaking English to complete study activities). Of the 141 participants who were assessed for eligibility, 53 participants were excluded from the study because they declined to participate (*n* = 42), did not meet inclusion criteria (e.g., HbA1c < 7; did not reside with the parent, or had no smartphone access; *n* = 11). Parents provided informed consent and adolescents provided assent. This study was approved by the Institutional Review Board at the University of Michigan (IRB-MED), approval number HUM00148853.

Participants completed a single data collection visit where all EF and T1D management measures were assessed. All measures were administered in person by a trained research assistant. Within 2 weeks after this visit, participants visited a university-affiliated lab for a blood draw to measure HbA1c.

### 2.2. Measures

#### 2.2.1. Executive Functioning (EF)

Adolescents and their parents both completed the Behavior Rating Inventory of Executive Functioning, 2nd Edition (BRIEF-2), a standardized, widely used, questionnaire-based executive functioning (EF) measure for children and adolescents [20]. The BRIEF-2 measures three main domains of EF: Behavior Regulation, Emotion Regulation, and Cognitive Regulation, which are summarized to calculate a Global Executive Composite (GEC) score indicating overall EF difficulties on a standardized scale. The GEC yields T-scores from 30 to 90 (higher scores = greater EF difficulties; Parent GEC Cronbach *α* = 0.97; Youth GEC Cronbach *α* = 0.95). BRIEF-2 T-scores from 65 to 69 are considered potentially clinically elevated, and T-scores at or above 70 are considered clinically elevated.

Adolescents completed behavioral measures of two EF components that were the focus of the intervention trial as they were hypothesized to be helpful in T1D management, specifically working memory and inhibitory control. Working memory was assessed using an in-person Forward/Backward Digit Span task from the Wechsler Intelligence Scale for Children (WISC), in which participants repeat back a sequence of numbers in either the order that was read to them, or in the reverse order [26]. The Forward Digit Span and Backward Digit Span tasks each consisted of eight items with two trials each. A correct response was worth 1 point, resulting in a maximum total score of 16 for each of the Forward Digit Span and Backward Digit Span; higher scores indicate stronger EF. Inhibitory control was assessed with a computer-based Go/No-Go task using a platform built to deliver such tasks in a reproducible manner (The Experiment Factory, https://expfactory-experiments.github.io/go-nogo/; The Experiment Factory, https://expfactory-experiments.github.io/go-nogo/; [27]. The Go/No-Go task is scored using reaction time (milliseconds) and accuracy (0%–100%); faster reaction time and greater accuracy indicate stronger EF. Research assistants were trained to administer the tasks by team members with experience in child assessment and in consultation with task developers.

#### 2.2.2. T1D Management

Adolescents and parents each reported on T1D management behaviors and parents reported on who takes responsibility for T1D management in the family.

##### 2.2.2.1. T1D Management Behaviors: Self-Care Inventory (SCI)

The Self-Care Inventory-Revised (SCI-R) is a 15-item questionnaire assessing diabetes-related self-care behaviors for individuals with either type 1 or type 2 diabetes [28]. The SCI-R is an updated version of the original SCI, modified to better reflect current diabetes practice [28]. Items cover the primary components of a diabetes treatment regimen such as monitoring and recording glucose levels, administering and adjusting insulin, regulating meals and exercise, and keeping medical appointments. Respondents indicate how often they do each behavior using a 5-point Likert scale (1 = “never do it”; 5 = “always do this as recommended, without fail”, with a “not applicable” option only for certain questions). Scores on the SCI-R are converted to a 0- to 100-point scale (higher scores = more optimal T1D management behaviors; parent Cronbach *α* = 0.80; youth Cronbach *α* = 0.76). Youth reported on their own behaviors; parents reported on youth behaviors.

##### 2.2.2.2. Responsibility for T1D Management: Diabetes Family Responsibility Questionnaire (DFRQ)

Parents completed the DFRQ to assess the degree of youth self-management of T1D. Parents rated 17 items (e.g., Noticing the early signs of an insulin reaction; Checking expiration dates on medical supplies; Telling teachers about diabetes) to indicate whether the item was mostly parent responsibility; parent and child equal responsibility, or mostly child responsibility. The DFRQ is scored such that lower values indicate that the parent takes more responsibility for diabetes care, whereas higher scores indicate the youth takes more responsibility for handling T1D responsibilities [29].

### 2.3. Diabetes Distress

#### 2.3.0.1. Problem Areas In Diabetes (PAID-T)

Youth reported their diabetes-specific distress using the Problem Areas In Diabetes-Teen (PAID-T) Scale [30]. Youth rated 14 items to indicate how much each item was a problem for them (e.g., Feeling overwhelmed by my diabetes regimen; Feeling that my friends or family act like “diabetes police”). Items were rated from 1 = not a problem to 6 = serious problem and summed to generate a total, scored such that higher scores indicate more diabetes-related distress. PAID-T scores of >44 are considered clinically elevated [31].

### 2.4. Demographic and T1D Variables

Parents reported on youth demographic characteristics including age (in years), sex assigned at birth (male or female), and race/ethnicity (White, Non-Hispanic/Latino (a); Black, Non-Hispanic/Latino (a); American Indian/Alaska Native; Asian/Pacific Islander; Hispanic/Latino (a) any race; Biracial/Other). Parents also reported their relationship to the adolescent (Biological mother; Biological father; Adoptive mother; Other), their own highest level of education completed, and family income range, as well as whether the adolescent used a continuous glucose monitor (CGM) and/or insulin pump. No data were available regarding closed-loop systems. HbA1c was used as an indicator of average blood glucose over the past 2–3 months. Results are expressed as % rounded to one decimal place; higher HbA1c levels reflect poorer glycemic control.

### 2.5. Statistical Analysis

Descriptive statistics were used to characterize demographic and T1D-related variables (youth age, sex, race/ethnicity; family income range, youth uses a CGM, youth uses an insulin pump, HbA1c level) and variables measuring youth EF, T1D management, and diabetes distress. Pearson correlation analyses were conducted to examine bivariate associations among EF, T1D management, and diabetes distress variables. Multivariable regression analyses were conducted to test associations between EF predictors and dependent variables indicating T1D management and diabetes distress. A total of four models were conducted predicting the following outcomes: youth self-reported SCI-R, parent-reported youth SCI-R, parent-reported DFRQ, and youth self-reported diabetes distress (PAID-T). Youth age, sex, and whether or not data collection had been conducted prior to or during the COVID-19 pandemic were tested as possible covariates. Only age was associated with key outcome variables and thus included as a covariate in the regression models. Youth self-reported and parent-reported youth BRIEF-2 Global Executive Composite (GEC) T-scores were used as the two EF questionnaire-based measures. In each model, the four performance-based EF variables were included: Forward Digit Span, Backward Digit Span, Go/No-Go reaction time, and Go/No-Go accuracy. A two-tailed alpha of <0.05 was used to indicate statistical significance for all analyses. The *p*-value and the magnitude of the effect size (small, medium, large) are reported.

## 3. Results

### 3.1. Descriptive Results

Descriptive statistics for all study variables are in Table 1. Mean age of youth in our sample was 15.44 ± 1.38 years; 46.6% were female. The sample was predominately White, with 81.8% of youth identifying as White, Non-Hispanic Latino (a). Median family income range was $100,000−199,999. Highest level of education completed for the responding parent ranged from completed high school/some college to graduate degree. Mean youth HbA1c was 8.6%. Parents reported that 72.4% of youth used an insulin pump and 83.0% used a CGM. Mean BRIEF-2 GEC T-Score was 52.43 ± 9.31 for youth self-report and 52.22 ± 8.97 for parent report. In our sample, youth-reported GEC was potentially clinically elevated (T-score ≥ 65) for 10.2% of youth (4.6% reporting clinically elevated GEC T-scores of ≥70). Parent-reported GEC was potentially clinically elevated for 8.0% of youth, and clinically elevated for 4.5% of youth. For diabetes distress, 22.5% of youth reported clinical levels of distress on the PAID-T.

### 3.2. Bivariate Associations Between EF, T1D Management, and Diabetes Distress Outcomes

Bivariate Pearson correlation results are presented in Table 2.

EF Indicators. BRIEF-2 GEC scores were not associated across reporters. Youth-reported BRIEF-2 scores did not associate with any performance-based measures. Youth Digit Span was associated with parent-reported BRIEF-2 GEC such that greater parent-reported youth EF difficulty correlated with worse Digit Span performance. Youth Go/No-Go accuracy was associated with parent-reported BRIEF-2 GEC, such that greater youth EF difficulties as reported by parents were associated with lower Go/No-Go accuracy (Table 2).

EF and T1D Management. Greater youth EF difficulties were associated with less-optimal T1D management (SCI-R) but only within reporters. There were no associations between performance-based EF measures and T1D management (Table 2).

EF and Diabetes Distress. Youth-reported BRIEF-2 GEC was associated with PAID-T scores such that poorer self-reported EF was associated with higher self-reported diabetes distress. Parent-reported BRIEF-2 GEC was not significantly associated with youth-reported diabetes distress. There were no associations between performance-based EF and youth-reported diabetes distress (Table 2).

HbA1c. Poorer youth-reported T1D management and more youth-reported diabetes distress associated with higher HbA1c. No EF variables correlated with HbA1c levels (Table 2).

### 3.3. Regression Analyses

Results of the multivariable regression analyses testing associations between EF predictors and T1D management and diabetes distress outcomes are presented in Table 3.

The overall model predicting youth self-reported T1D management on the SCI-R was significant (*F* = 3.20 (7,74), *p*  < 0.005), explaining 23% of the variance in SCI-R scores (Table 3). Youth self-reported BRIEF-2 GEC was a significant individual predictor, such that greater self-reported EF difficulties were associated with lower self-reported T1D management (*β* = −0.40, *p*=0.001, moderate effect size (es)). Neither parent-reported EF nor the performance-based EF measures were significant individual predictors of youth self-reported T1D management on the SCI-R.

The overall model predicting parent-reported youth T1D management on the SCI-R was not significant (*F* = 1.89 (7,74), *p*=0.08; see Table 3). Parent-reported youth BRIEF-2 GEC was a significant individual predictor in the NS model, such that greater EF difficulties as measured on the BRIEF-2 were associated with lower parent-reported youth T1D management (*β* = −0.33, *p*=0.01, small es). Neither youth self-reported EF nor performance-based EF measures were significant individual predictors of parent-reported T1D management on the SCI-R.

The overall model predicting parent-reported responsibility for diabetes management on the DFRQ was significant (*F* = 3.73 (7,74), *p*  < 0.002), explaining 26% of the variance in DFRQ scores (see Table 3). Youth age and go/no-go performance were significant individual predictors, such that older age (*β* = 0.43, *p* < 0.001, moderate es), longer (slower) reaction time (*β* = 0.23, *p*=0.02, small es), and poorer accuracy (*β* = −0.23, *p*=0.04, small es) were associated with more youth responsibility for management as reported by parents on the DFRQ. Neither parent- or youth-reported EF nor other performance-based EF measures were significant individual predictors of parent-reported responsibility for T1D management.

The overall model predicting youth-reported diabetes distress on the PAID-T was significant (*F* = 3.43 (7,66), p  < 0.004), explaining 26% of the variance in PAID-T scores (see Table 3). Youth self-reported BRIEF GEC was a significant individual predictor, such that greater difficulties on the BRIEF were associated with more diabetes distress (*β* = 0.41, *p* < 0.001, moderate es). Neither parent-reported youth EF nor the performance-based EF measures were significant individual predictors of youth-reported diabetes distress on the PAID-T.

## 4. Discussion

The current study had three main findings that contextualize our understanding of EF, T1D management, and diabetes distress, as well as EF assessment in youth more broadly. First, youth self-reported EF was associated with youth self-reported diabetes distress. Second, within-reporter associations were strong: youth self-reported EF predicted self-reported but not parent-reported T1D management, whereas parent reports of youth EF predicted parent-reported but not youth self-reported T1D management. Bivariate analyses also showed that youth self-reports and parent reports of T1D management behaviors were correlated, whereas youth and parent reports of EF were not. Third, questionnaire-based and performance-based assessments of EF were generally not associated. Other findings were that older age, but poorer performance on a go/no-go task predicted greater youth responsibility for T1D management. Findings are discussed below with regard to implications for future research and clinical efforts.

### 4.1. EF and Diabetes Distress

As EF supports emotion regulation skills, we had hypothesized that EF difficulties would be positively associated with youth diabetes distress. This hypothesis was supported, with moderate effect sizes found for youth self-reported EF. Youth-reported EF difficulty was associated with greater diabetes distress on the PAID-T. Diabetes distress is an important indicator of well-being among youth with T1D, with greater diabetes distress associated with poorer diabetes-related outcomes [2, 15, 16]. Prior work has not examined EF and diabetes distress in youth. One study found that greater parent-reported youth EF difficulties were associated with poorer youth-reported diabetes-related quality of life, but diabetes distress was not measured [9]. Weaker EF skills can make it difficult for adolescents to recognize and regulate their emotions, possibly disrupting T1D management. For example, an inability to manage diabetes distress effectively can result in avoiding T1D management needs [16]. In contrast, the capacity to recognize and engage in intentional strategies to address negative emotions when they occur could reduce diabetes distress and prompt more effective T1D management [18, 32]. The current study starts to fill a gap in data on EF and diabetes distress; mapping the different pathways through which EF skills could reduce diabetes distress by supporting emotion regulation and/or by promoting T1D management will be important in future work.

Parents' reports of their child's overall EF difficulties on the BRIEF did not associate with youth-reported diabetes distress in regression models. A recent study examining concordance in parent and youth reports found that distress around diabetes had implications for T1D-related conflict, with youth distress, compared to parent distress most predictive of conflict [33]. Cross-informant differences in perceived internal states like distress are important to keep in mind when considering how information is gathered in the clinic setting, particularly across the adolescent period as the adolescent transitions to become the primary informant [34]. Although current results emphasize the importance of adolescents' views of their own functioning, parents' perspectives likely remain essential for understanding overall emotional dynamics at the family level and implications of these for addressing diabetes distress.

### 4.2. EF and T1D Management

We found that EF difficulties and T1D self-management were negatively associated, as hypothesized, with strongest concordance within the reporter. Furthermore, although parent and youth reports of EF on the BRIEF were not correlated in bivariate analyses, parent and youth reports of T1D management behaviors were positively correlated, suggesting some cross-reporter concordance. Compared to EF, T1D management may have more concrete indicators that can be externally observed, leading to greater agreement between youth and parents. Yet, the agreement was still not perfect, suggesting that reports from both youth and parents on EF and T1D management could each yield unique information.

Comparable to prior work on EF and T1D management [22], we found moderate effect sizes for youth reports and smaller effect sizes for parent reports of EF-T1D management associations. Our findings of greater within- than across-reporter concordance are not surprising and could be due in part to reporter bias. It may also reflect that parents and youth increasingly have access to different samples of behavior across early to later adolescence, so multiple perspectives are valuable. Youth can provide unique and direct insight into both EF difficulties and T1D management, while parents have increasingly limited opportunities to observe such behaviors directly as youth begin to spend more time in non-familial contexts and take on more independent T1D management, with varying success [35]. Yet, parents likely perceive patterns in both EF and T1D management behaviors that can affect T1D outcomes over time that youth may not always recognize (e.g., capacity to engage in long-term planning).

Results are also consistent with previous findings that greater EF skills are associated with more optimal T1D management when EF is measured through questionnaire-based measures, but not when EF is measured through performance-based measures [19]. To our knowledge, only one other study has used both questionnaire-based and performance-based measures of EF to examine EF and T1D management across the adolescent period [25] and also found that parent-reported EF difficulties, not performance-based EF, associated with poorer parent-reported T1D management in youth. This study did not examine youth self-reports. Prior work with older adolescents did find that youth self-reported, but not performance-based EF, was associated with T1D management, but these studies did not include parent reports [21, 23].

### 4.3. Different Approaches to EF Assessment

The current study thus extends prior research by including performance-based, youth self-reports, and parent-reported EF measures. Similar to prior work, we found that self-reported EF were more strongly related than performance-based measures to adolescents' T1D management. It is reasonable to consider what different measurement approaches that purport to assess a singular construct, EF, are actually assessing. Whereas performance-based EF measures assess specific cognitive skills reflecting capacity in structured tasks (“state-like” EF), parent and youth reports reflect how EF functions on an everyday basis (“trait-like” EF), reflecting capacity across contexts and time [36]. These different approaches to measurement may also indicate a competence–performance distinction such that performance-based measures allow demonstration of a given skill when in a controlled context, whereas competence in that skill may not be demonstrated in “real-world” contexts where more distractions are present. We observed that performance-based measures of “state-like” EF were not correlated with “trait-like” EF. Furthermore, the BRIEF directly queries about EF-related difficulties in daily life. Therefore, an adolescent's capacity to use their EF skills to cope with challenges, as assessed on the BRIEF, may functionally differ from task-based assessment of EF capacity in the absence of contextual challenges.

Prior work also suggests that questionnaire-based and performance-based measures of EF generally assess different aspects of cognitive functioning, and the strengths and limitations of each approach measures should be considered in interpretation [37]. Performance-based measures of EF assess the algorithmic mind, a level of analysis primarily concerning the efficiency of cognitive processing [37]. However, performance-based measures of EF raise issues of task impurity and test–retest reliability [38, 39], which may affect correlations not only between different EF tasks but also associations of EF tasks with clinical outcomes like T1D management. Task impurity refers to performance-based EF measures capturing not just the EF construct of interest, but lower-order processes specific to the task (e.g., attention, processing speed, motor control) [39]. To reduce this issue, multiple performance-based tasks that utilize different lower-order cognitive processes could be used to create composite scores for EF constructs in future research. Additionally, given that performance-based measures of EF are completed in a structured setting at a specific time, variations in individual or contextual factors also may lead to greater intra-individual variability in EF performance [36]. For example, relevant to our sample, fluctuations in blood glucose can affect cognitive performance [36, 40]. Blood glucose was not measured prior to EF tasks in the current study, though this may be valuable to do in future work.

In contrast to performance-based assessments, questionnaire-based measures assess the reflective mind, which relates to beliefs and actions relevant to goal pursuit [37]. Metacognition, which is assessed on the BRIEF, describes the ability to reflect on one's own thoughts and behaviors in this way. Metacognitive capacity develops throughout adolescence and has an important role in self-monitoring of behaviors, which is highly relevant for T1D management. Compared to performance-based EF measures, questionnaire-based assessments from both youth and parents may better indicate how EF skills shape everyday behaviors required to manage T1D that involve integrating multiple cognitive and behavioral tasks to achieve a desired goal, perhaps driving the competence-performance distinction in the T1D management context. Our findings that performance-based and questionnaire-based EF measures were not consistently correlated aligns with previous research suggesting that these approaches to measurement assess distinct components of EF but may offer complementary insight when used together [36, 41]. Regarding differences across informants, youth self-reported EF did not correlate with any performance-based EF tasks, but parent-reported EF was positively associated with performance-based digit span tasks in bivariate analyses. This suggests parents may perceive their child's EF abilities differently than their child does and perhaps, that parents may have insight into their child's EF skills and the algorithmic mind as captured through performance-based EF measures. Future research should strive to improve precision in EF measurement by developing and testing integrative methods that capture both cognitive skills assessed in performance-based tasks, and the applied, real-world aspects of EF functioning that are captured by questionnaire measures from multiple informants.

### 4.4. Developmental Considerations

Regarding development, prior research suggests that EF begins as a unitary construct in early childhood, and develops into several related, but distinct constructs throughout the adolescent period [42–45]. Due to such different rates of development for separate aspects of EF (inhibition, shifting, and working memory), these constructs may not always be strongly correlated during adolescence. Such developmental changes may in part contribute to discrepancies in performance across different EF tasks as well as between performance-based and questionnaire-based measures. Further research is needed to establish how EF structure changes across adolescence, and what specific aspects of adolescent development affect the rate of change in specific EF skills.

We also noted developmental considerations specific to T1D management in the family context. Our regression models suggested that older child age predicted greater child responsibility for T1D management (moderate effect size). These age effects make sense and may reflect parents' perceptions of their child's maturity and readiness to take on more independent T1D management as they get older. The regression analysis results also showed that poorer go/no-go task performance—specifically longer response times and less accurate responses—predicted greater child responsibility for T1D management (small effect sizes), however, which was somewhat puzzling. It could be that regardless of accuracy, parents may perceive a child's quick response time in a task like this as indicating some level of or propensity toward impulsivity that is important for driving decision-making around responsibility for T1D management. That is, parents may focus primarily on a child's speed of response and value slower responses, as seen here, if they feel the stakes are too high to tolerate impulsivity which may lead to inaccuracies with diabetes-related tasks, as extreme blood glucose excursions can have life-threatening impacts. This notion would support parents' perhaps over-prioritizing the need for less impulsivity over potential accuracy, preferring the child to move slowly and cautiously to reduce the chance for any potential errors when they are managing diabetes.

In short, multiple factors likely influence the balance of parent compared to youth management of T1D across this developmental period. Although EF skills undergo considerable developmental change during this time, EF skills may play some role in indicating child readiness to assume responsibility for T1D management, such associations are emergent and we cannot determine the full nature or direction of these associations given the cross-sectional design of the current study. These questions would be helpful to examine in future longitudinal work. Prior research has found that parents use age or pubertal maturation as a cue for adolescents' readiness to assume increased T1D management responsibilities [46, 47]. Yet, neither age nor pubertal maturation are always reliably associated with adequate skills for diabetes management, and prematurely decreasing parental management may contribute to suboptimal T1D management behaviors and inconsistent glycemic outcomes.

Considering the many physiological and psychological changes that influence both EF and T1D management across adolescence, it is important for families to consider how best to transition children to more independent T1D management while still providing ample scaffolding during this time of extended and significant developmental change. Adolescence is a period marked by developmental discontinuity, as noted above, with emotional reactivity often interfering with adolescents' implementation of growing EF capacities such as planning and organization [24]. It may be helpful for providers as well as parents to take an active role in illustrating how child skills across EF domains may map on to specific T1D management responsibilities and considering child factors when planning for transitions in care.

### 4.5. Implications

Understanding the role of EF in T1D management, and particularly how EF skills may moderate diabetes-related distress, can inform healthcare providers seeking to support T1D management behaviors and improve well-being for youth with T1D across adolescence. Understanding associations between EF and diabetes distress could have clinical implications for managing diabetes distress, possibly through targeting EF and emotion regulation skills [18]. Our results also suggest that parent and youth reflections on youth EF functioning may have more salient implications for understanding T1D management than performance-based EF, which measures context-specific cognitive capacities rather than planning of goal-oriented behaviors. This finding may also suggest that limitations in EF in a cognitive performance task may not necessarily lead to poorer T1D management behaviors. To improve T1D management by targeting EF, interventions that help adolescents learn and apply skills in the context of actual T1D management tasks may be more effective than EF-enhancing interventions that are unrelated to T1D management behaviors, such as memory training. Targets of such T1D EF skill-related interventions may include supply management, planning for scheduled CGM or pump changes, execution of hypo- and hyperglycemic event management practices, and anticipation of blood glucose excursions related to diet and exercise. Finally, gathering perspectives on both EF and T1D management behaviors from youth and parents across the entire adolescent period is likely critical for developmentally sensitive interventions, as youth undergo developmental changes that impact both their EF skills and their capacities to perceive and report on any challenges as they assume increased self-management of T1D across the adolescent transition.

## 5. Strengths and Limitations

Strengths of the current study were that we examined associations between EF and diabetes distress as well as T1D management behaviors, and that we used both questionnaire-based and performance-based measures of youth EF to assess whether different methods of measuring EF yield different associations between EF and our outcomes of interest. We collected reports of youth EF and T1D management from both youth and parents, allowing us to explore these different associations. We acknowledge limitations, including our cross-sectional design and sample of youth participating in an intervention study, who were also predominately from high-income families, with highly educated parents, and mostly White. Studies have shown positive correlations between family income and EF [48], and the sample had low rates of clinical EF difficulties, although similar to other T1D studies [6] and with comparable rates of diabetes distress as in prior work [33]. These sample considerations create cautions for generalizability. As noted above, we did not check blood glucose during the study protocol prior to the EF tasks to reduce burden and study visit length. Participants were encouraged to follow their typical diabetes care throughout the visit, including responding to blood sugar excursions, and many of the youth (83%) were using CGMs. As part of the informed consent/assent procedures and throughout the visit, we checked in with the children and provided opportunities for them to eat a snack or take breaks as needed. Preintervention assessments were also collected during the COVID-19 pandemic for about one-half of the sample. Although the timing of data collection in relation to pandemic onset was tested as a covariate and found not to have significant effects, it is certainly possible that pandemic-related individual and environmental factors could have influenced family experiences and thus also some of our data and findings.

## 6. Conclusions

For youth with T1D, adolescence is a period of increased risk for T1D management challenges and suboptimal glycemic outcomes for a host of reasons. Promoting effective diabetes management in adolescence is critical for improving health outcomes and preparing adolescents to manage T1D independently. Findings suggested that EF skills are important not only for T1D management behaviors but also for diabetes distress. Different measurement approaches yielded unique information; understanding parent and youth perspectives on EF challenges may be more useful than youths' performance-based EF skills in supporting T1D management behaviors and reducing diabetes distress. Promoting EF in the context of T1D management tasks, as well as providing broader support to manage well-being and distress around diabetes may be particularly helpful as families navigate the developmental period of adolescence.

## Figures and Tables

**Table 1:** Participant characteristics.

Variable	M (SD) or *N* (%)
Demographic and T1D characteristics	
Youth age (years)	15.44 (1.38)
Youth is female	41 (46.6%)
Youth race/ethnicity	
White, non-Hispanic/Latino (a)	72 (81.8%)
Black, non-Hispanic/Latino (a)	5 (5.7%)
Other/Biracial, non-Hispanic/Latino (a)	4 (4.5%)
American Indian/Alaska Native or Asian/Pacific Islander, non-Hispanic/Latino (a)	2 (2.3%)
Hispanic/Latino (a) any race	2 (2.3%)
Missing/declined to respond	3 (3.4%)
Youth uses CGM	73 (83.0%)
Youth uses insulin pump	63 (72.4%)
Youth HbA1c	8.61 (1.37)
Parent respondent is	
Biological mother	75 (85.2%)
Biological father	7 (8.0%)
Adoptive mother	3 (3.4%)
Other	3 (3.4%)
Median family income range ($)	100,000−199,999
Participating parent's highest level of education	
Completed high school/some college or post-high school training	33 (37.5%)
Completed college	29 (33.0%)
Completed advanced degree	26 (29.5%)
Study variables	
Youth-reported BRIEF-2 Global Executive Composite T-score	52.43 (9.31)
Parent-reported BRIEF-2 Global Executive Composite T-score	52.22 (8.97)
Youth Forward Digit Span	10.15 (2.45)
Youth Backward Digit Span	7.95 (2.25)
Youth Go/No-Go Reaction Time (ms)	364.58 (42.39)
Youth Go/No-Go Accuracy (%)	97.00 (2.50)
Youth-reported SCI-R	72.05 (16.78)
Parent-reported SCI-R	66.20 (15.83)
Youth-reported PAID-T	31.89 (16.41)
Parent-reported DFRQ	33.45 (5.48)

Abbreviations: BRIEF, Behavior Rating Inventory of Executive Functioning; CGM, continuous glucose monitor; DFRQ, Diabetes Family Responsibility Questionnaire; PAID-T, Problem Areas In Diabetes-Teen; SCI-R, Self-Care Inventory-Revised; T1D, type 1 diabetes.

**Table 2:** Bivariate correlation analyses for executive functioning, T1D management, diabetes distress, and HbA1c.

Variable	1	2	3	4	5	6	7	8	9	10
Executive functioning										
1. Youth BRIEF-2 Global Executive Composite	—	—	—	—	—	—	—	—	—	—
2. Parent BRIEF-2 Global Executive Composite	0.13	—	—	—	—	—	—	—	—	—
3. Youth Forward Digit Span	−0.04	−0.25^*∗*^	—	—	—	—	—	—	—	—
4. Youth Backward Digit Span	0.04	−0.22^*∗*^	0.43^*∗∗*^	—	—	—	—	—	—	—
5. Youth Go/No-Go Reaction Time	−0.02	0.06	−0.14	−0.04	—	—	—	—	—	—
6. Youth Go/No-Go Accuracy	0.07	−0.24^*∗*^	0.22^*∗*^	0.20	0.14	—	—	—	—	—
T1D Management, Diabetes Distress										
7. Youth SCI-R	−0.44^*∗∗*^	−0.08	−0.01	−0.08	0.08	−0.05	—	—	—	—
8. Parent SCI-R	−0.01	−0.25^*∗*^	−0.09	−0.01	0.20	−0.05	0.33^*∗∗*^	—	—	—
9. Parent DFRQ	0.14	−0.11	0.01	0.19	0.12	−0.04	−0.12	0.18	—	—
10. Youth PAID-T	0.44^*∗∗*^	0.20	−0.06	0.07	0.19	0.04	−0.35^*∗*^	−0.11	0.18	—
11. HbA1c Level	0.06	0.10	−0.15	−0.13	0.17	−0.04	−0.26^*∗*^	0.14	0.34^*∗*^	−0.02

Abbreviations: BRIEF, Behavior Rating Inventory of Executive Functioning; DFRQ, Diabetes Family Responsibility Questionnaire; HbA1c, hemoglobin A1c; PAID-T, Problem Areas In Diabetes-Teen; SCI-R, Self-Care Inventory-Revised; T1D, type 1 diabetes.

*⁣* ^*∗*^ *p* < 0.05; *⁣*^*∗∗*^*p* < 0.01.

**Table 3:** Regression analyses predicting T1D management and diabetes distress.

Step and predictor variable	B	SEB	*β*	F (df)	*R* ^2^
Youth-reported SCI-R					
Youth age	−1.55	1.01	−0.17	3.20 (7,74)*⁣*^*∗*^	0.23
Youth BRIEF-2 GEC	−0.55	0.15	−0.40^*∗∗*^	—	—
Parent BRIEF-2 GEC	−0.05	0.16	−0.04	—	—
Forward Digit Span	−0.10	0.61	−0.02	—	—
Backward Digit Span	−0.31	0.65	−0.06	—	—
Go/No-Go Reaction Time	0.01	0.03	0.04	—	—
Go/No-Go Accuracy	5.63	57.16	0.01	—	—
Parent-reported SCI-R	—	—	—	1.89 (7.74)	0.15
Youth age	−0.91	1.17	−0.09	—	—
Youth BRIEF-2 GEC	0.10	0.17	0.07	—	—
Parent BRIEF-2 GEC	−0.51	0.18	−0.33^*∗*^	—	—
Forward Digit Span	−0.65	0.71	−0.11	—	—
Backward Digit Span	0.00	0.76	−0.00	—	—
Go/No-Go Reaction Time	0.07	0.04	0.21	—	—
Go/No-Go Accuracy	−66.75	66.00	−0.12	—	—
Parent-reported DFRQ	—	—	—	3.73 (7,74)*⁣*^*∗∗*^	0.26
Youth age	1.70	0.43	0.43^*∗∗*^	—	—
Youth BRIEF-2 GEC	0.05	0.06	0.08	—	—
Parent BRIEF-2 GEC	−0.11	0.07	−0.17	—	—
Forward Digit Span	−0.02	0.26	−0.01	—	—
Backward Digit Span	0.44	0.28	0.18	—	—
Go/No-Go Reaction Time	0.03	0.01	0.23^*∗*^	—	—
Go/No-Go Accuracy	−50.65	24.02	−0.23^*∗*^	—	—
Youth-reported PAID-T	—	—	—	3.33 (7,66)*⁣*^*∗*^	0.26
Youth age	0.63	1.35	0.05	—	—
Youth BRIEF-2 GEC	0.72	0.19	0.41^*∗*^	—	—
Parent BRIEF-2 GEC	0.27	0.21	0.15	—	—
Forward Digit Span	−0.11	0.82	−0.02	—	—
Backward Digit Span	0.70	0.87	0.10	—	—
Go/No-Go Reaction Time	0.08	0.04	0.20	—	—
Go/No-Go Accuracy	−1.56	76.09	0.00	—	—

*Note:* The SCI-R is scored such that higher scores indicate better T1D management; DFRQ such that higher scores indicate that youth takes more responsibility; PAID-T such that higher scores indicate greater diabetes distress.

Abbreviations: BRIEF, Behavior Rating Inventory of Executive Functioning; DFRQ, Diabetes Family Responsibility Questionnaire; PAID-T, Problem Areas In Diabetes-Teen; SCI-R, Self-Care Inventory-Revised; T1D, type 1 diabetes.

*⁣* ^*∗*^ *p* < 0.05; *⁣*^*∗∗*^*p* < 0.01.

## Data Availability

The data that support the findings of this study are available from the corresponding author upon reasonable request.
